# Crystal structure of 4-hy­droxy-3-meth­oxy­benzaldehyde 4-methyl­thio­semi­carbazone methanol monosolvate

**DOI:** 10.1107/S2056989015007227

**Published:** 2015-04-18

**Authors:** Adriano Bof de Oliveira, Johannes Beck, Christian Landvogt, Bárbara Regina Santos Feitosa

**Affiliations:** a Departamento de Química, Universidade Federal de Sergipe, Av. Marechal Rondon s/n, 49100-000 São Cristóvão-SE, Brazil; b Institut für Anorganische Chemie, Rheinische Friedrich-Wilhelms-Universität Bonn, Gerhard-Domagk-Strasse 1, D-53121 Bonn, Germany

**Keywords:** crystal structure, bifurcated hydrogen bond, thio­semicarbazone derivative from natural product (vanillin)

## Abstract

In the title solvate, C_15_H_15_N_3_O_2_S·CH_3_OH, the thio­semicarbazone mol­ecule is approximately planar; the maximum deviation from the mean plane is 0.4659 (14) Å and the dihedral angle between the aromatic rings is 9.83 (8)°. This conformation is supported by an intra­molecular N—H⋯N hydrogen bond. In the crystal, the thio­semicarbazone mol­ecules are linked into dimers by pairs of N—H⋯S hydrogen bonds, thereby generating *R*_2_^2^(8) loops. The methanol solvent mol­ecule bonds to the thio­semicarbazone mol­ecule through a bifurcated O—H⋯(O,O) hydrogen bond and also accepts an O—H⋯O link from the thio­semicarbazone mol­ecule. Together, these links generate a three-dimensional network.

## Related literature   

For one of the first reports of thio­semicarbazone derivatives synthesis, see: Freund & Schander (1902 ▸). For the report concerning the synthesis and crystal structure of 4-hy­droxy-3-meth­oxy­benzaldehyde 4-phenyl­thio­semicarbazone, see: Oliveira *et al.* (2014 ▸).
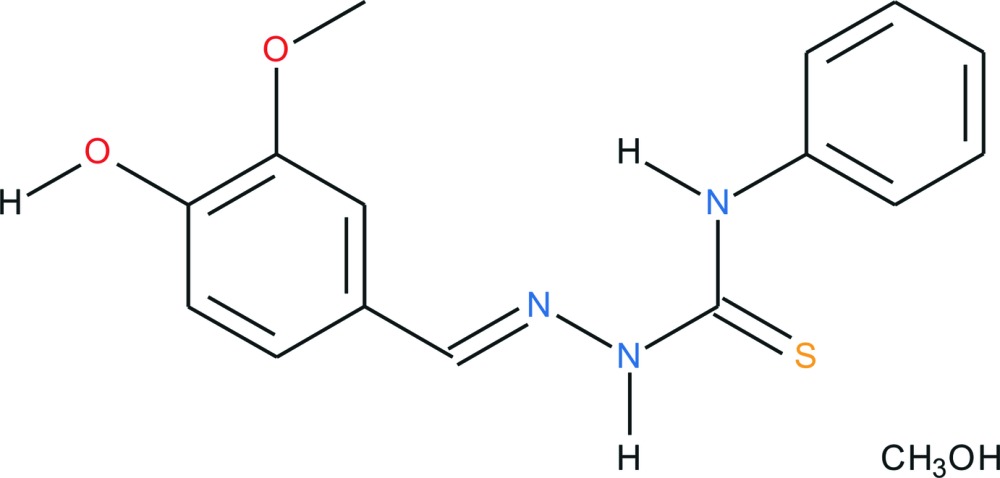


## Experimental   

### Crystal data  

C_15_H_15_N_3_O_2_S·CH_4_O
*M* *_r_* = 333.40
Monoclinic, 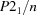

*a* = 11.1833 (2) Å
*b* = 8.4207 (2) Å
*c* = 17.2521 (4) Å
β = 95.752 (1)°
*V* = 1616.47 (6) Å^3^
*Z* = 4
Mo *K*α radiation
μ = 0.22 mm^−1^
*T* = 123 K
0.29 × 0.15 × 0.09 mm

### Data collection  

Nonius KappaCCD diffractometer
Absorption correction: multi-scan (Blessing, 1995 ▸) *T*_min_ = 0.924, *T*_max_ = 0.983
46461 measured reflections
3692 independent reflections
2926 reflections with *I* > 2σ(*I*)
*R*_int_ = 0.053

### Refinement  

*R*[*F* ^2^ > 2σ(*F* ^2^)] = 0.034
*wR*(*F* ^2^) = 0.091
*S* = 1.05
3692 reflections
284 parameters
All H-atom parameters refined
Δρ_max_ = 0.21 e Å^−3^
Δρ_min_ = −0.32 e Å^−3^

### 

Data collection: *COLLECT* (Nonius, 1998 ▸); cell refinement: *SCALEPACK* (Otwinowski & Minor, 1997 ▸); data reduction: *DENZO* (Otwinowski & Minor, 1997 ▸) and *SCALEPACK*; program(s) used to solve structure: *SUPERFLIP* (Palatinus & Chapuis, 2007 ▸); program(s) used to refine structure: *SHELXL2013* (Sheldrick, 2015 ▸); molecular graphics: *DIAMOND* (Brandenburg, 2010 ▸); software used to prepare material for publication: *publCIF* (Westrip, 2010 ▸) and *WinGX* (Farrugia, 2012 ▸).

## Supplementary Material

Crystal structure: contains datablock(s) I, publication_text. DOI: 10.1107/S2056989015007227/hb7402sup1.cif

Structure factors: contains datablock(s) I. DOI: 10.1107/S2056989015007227/hb7402Isup2.hkl

Supporting information file. DOI: 10.1107/S2056989015007227/hb7402Isup3.cml

**Fig. 1.**
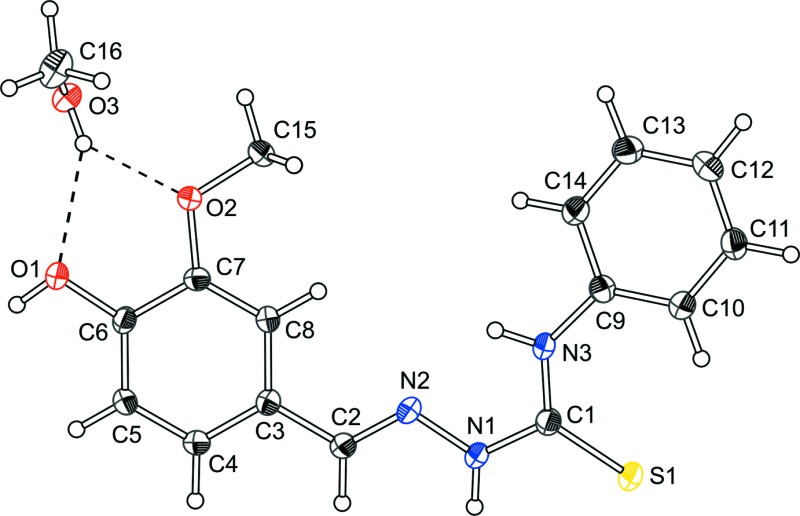
: The molecular structure of the title compound with displacement ellipsoids drawn at the 50% probability level. H atoms are drawn isotropically. The bifurcated hydrogen bonds are shown as dashed lines.

. DOI: 10.1107/S2056989015007227/hb7402fig1.tif
The mol­ecular structure of the title compound with displacement ellipsoids drawn at the 50% probability level. H atoms are drawn isotropically. The bifurcated hydrogen bonds are shown as dashed lines.

**Fig. 2.**
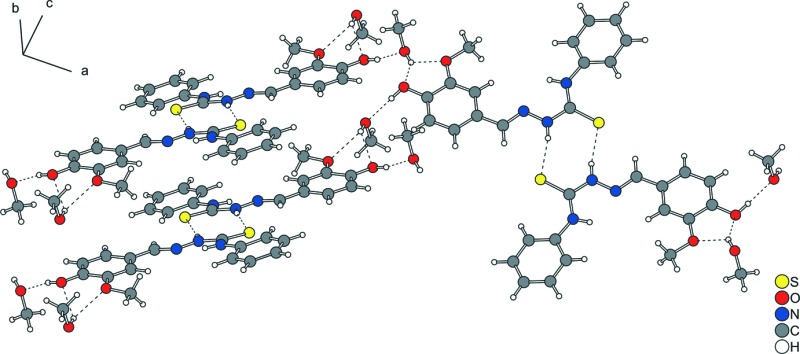
: View of the hydrogen bonding in the structure of the title compound showing the three dimensional H-bonded network. Hydrogen bonding is shown as dashed lines.

. DOI: 10.1107/S2056989015007227/hb7402fig2.tif
View of the hydrogen bonding in the structure of the title compound showing the three dimensional H-bonded network. Hydrogen bonding is shown as dashed lines.

CCDC reference: 1059141

Additional supporting information:  crystallographic information; 3D view; checkCIF report

## Figures and Tables

**Table 1:** Hydrogen-bond geometry (, )

*D*H*A*	*D*H	H*A*	*D* *A*	*D*H*A*
N3H13N2	0.866(18)	2.082(17)	2.5865(16)	116.4(14)
N1H14S1^i^	0.896(19)	2.530(19)	3.4033(13)	165.2(15)
O1H15O3^ii^	0.86(2)	1.81(2)	2.6562(14)	167.7(19)
O3H19O2	0.83(2)	2.26(2)	2.8853(14)	132.1(18)
O3H19O1	0.83(2)	2.46(2)	3.1645(15)	144.2(18)

Symmetry codes: (i) 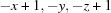; (ii) 
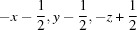
.

## supplementary crystallographic information

### S1. Structural commentary

Concerning our on-going research on the supra­molecular chemistry of thio­semicarbazone derivatives from natural products, we report herein the synthesis and structure of the 4-hy­droxy-3-meth­oxy­benzaldehyde 4-phenyl­thio­semicarbazone methanol monosolvate, a thio­semicarbazone derivative from vanillin. The thio­semicarbazone group of the title compound doesn't match the ideal planarity and the maximum deviation from the mean plane of the non-H atoms concerning the thio­semicarbazone group amounts to 0.4659 (14) Å for C15 and the dihedral angle between the two aromatic rings amounts to 9.83 (8)° (Fig. 1). In the crystal, molecules are linked into dimers *via* pairs of N1—H14···S1 hydrogen bonds. The dimers are linked into a three dimensional hydrogen bonded network through the methanol molecules by the O1—H15···O3, O3—H19···O2 and O3—H19···O1 hydrogen inter­actions (Fig. 2). In addition, one inter­molecular N3—H13···N2 hydrogen inter­action is also observed (Table 1).

The crystal structure of the solvate free 4-hy­droxy-3-meth­oxy­benzaldehyde 4-phenyl­thio­semicarbazone is already published (Oliveira *et al.*, 2014) and the molecules are linked by N—H···S hydrogen inter­actions into dimers. Additionally, the dimers are linked by N—H···O and O—H···S hydrogen inter­actions building a three-dimensional hydrogen-bonded network.

In the actual structure, the presence of the methanol solvate molecules maintains the dimensionality of the network. As the outstanding feature, a bifurcated hydrogen bond is observed. The atom H19 of the hy­droxy group of the methanol solvate builds a bifurcated hydrogen bond with the O1 and O2 atoms of the *ortho*-hy­droxy-meth­oxy entity of the thio­semicarbazone derivative. The H19···O2 and H19···O1 distances amount to 2.26 (2) Å and 2.46 (2) Å (Fig. 1). As the difference between the lengths of the two hydrogen inter­actions is about 0.2 Å, the bifurcation is considered symmetric. Due to the hydrogen-bond inter­actions promoted by the solvate molecule, the supra­molecularity of the structure modifies the arrangement molecules but the three-dimensional H-bonded network is preserved (Fig. 2).

### S2. Synthesis and crystallization

Starting materials were commercially available and were used without further purification. The synthesis of the title compound, 4-hy­droxy-3-meth­oxy­benzaldehyde-4-methyl­thio­semicarbazone, was adapted from a previously procedure (Freund & Schander, 1902 and Oliveira *et al.*, 2014). Colourless blocks were obtained unexpectedly from a mixture containing uranyl acetate dihydrate and the title compound in methanol by the slow evaporation of the solvent.

### S3. Refinement

Crystal data, data collection and structure refinement details are summarized in the Experimental part. All hydrogen atoms were localized in a difference density Fourier map. Their positions and isotropic displacement parameters were refined.

### Crystal data

**Table:**

C_15_H_15_N_3_O_2_S·CH_4_O	*F*(000) = 704
*M**_r_* = 333.40	*D*_x_ = 1.370 Mg m^−^^3^
Monoclinic, *P*2_1_/*n*	Mo *K*α radiation, λ = 0.71073 Å
Hall symbol: -P 2yn	Cell parameters from 32712 reflections
*a* = 11.1833 (2) Å	θ = 2.9–27.5°
*b* = 8.4207 (2) Å	µ = 0.22 mm^−^^1^
*c* = 17.2521 (4) Å	*T* = 123 K
β = 95.752 (1)°	Block, colorless
*V* = 1616.47 (6) Å^3^	0.29 × 0.15 × 0.09 mm
*Z* = 4	

### Data collection

**Table:**

Nonius KappaCCD diffractometer	3692 independent reflections
Radiation source: fine-focus sealed tube, Nonius KappaCCD	2926 reflections with *I* > 2σ(*I*)
Graphite monochromator	*R*_int_ = 0.053
Detector resolution: 9 pixels mm^-1^	θ_max_ = 27.5°, θ_min_ = 3.0°
CCD rotation images, thick slices scans	*h* = −14→14
Absorption correction: multi-scan (Blessing, 1995)	*k* = −10→10
*T*_min_ = 0.924, *T*_max_ = 0.983	*l* = −22→22
46461 measured reflections	

### Refinement

**Table:**

Refinement on *F*^2^	Primary atom site location: iterative
Least-squares matrix: full	Secondary atom site location: difference Fourier map
*R*[*F*^2^ > 2σ(*F*^2^)] = 0.034	Hydrogen site location: difference Fourier map
*wR*(*F*^2^) = 0.091	All H-atom parameters refined
*S* = 1.05	*w* = 1/[σ^2^(*F*_o_^2^) + (0.0462*P*)^2^ + 0.4527*P*] where *P* = (*F*_o_^2^ + 2*F*_c_^2^)/3
3692 reflections	(Δ/σ)_max_ < 0.001
284 parameters	Δρ_max_ = 0.21 e Å^−^^3^
0 restraints	Δρ_min_ = −0.32 e Å^−^^3^

### Special details

**Table:**

Geometry. All e.s.d.'s (except the e.s.d. in the dihedral angle between two l.s. planes) are estimated using the full covariance matrix. The cell e.s.d.'s are taken into account individually in the estimation of e.s.d.'s in distances, angles and torsion angles; correlations between e.s.d.'s in cell parameters are only used when they are defined by crystal symmetry. An approximate (isotropic) treatment of cell e.s.d.'s is used for estimating e.s.d.'s involving l.s. planes.
Refinement. Refinement of *F*^2^ against ALL reflections. The weighted *R*-factor *wR* and goodness of fit *S* are based on *F*^2^, conventional *R*-factors *R* are based on *F*, with *F* set to zero for negative *F*^2^. The threshold expression of *F*^2^ > σ(*F*^2^) is used only for calculating *R*-factors(gt) *etc*. and is not relevant to the choice of reflections for refinement. *R*-factors based on *F*^2^ are statistically about twice as large as those based on *F*, and *R*- factors based on ALL data will be even larger.

### Fractional atomic coordinates and isotropic or equivalent isotropic displacement parameters (Å^2^)

**Table:**

	*x*	*y*	*z*	*U*_iso_*/*U*_eq_	
S1	0.61038 (3)	0.16794 (5)	0.57257 (2)	0.03151 (12)	
O1	−0.20192 (9)	0.56394 (12)	0.31751 (6)	0.0250 (2)	
O2	−0.03515 (8)	0.71233 (11)	0.40440 (6)	0.0228 (2)	
O3	−0.17481 (9)	0.93717 (13)	0.30693 (6)	0.0267 (2)	
N1	0.39757 (10)	0.21003 (14)	0.49762 (7)	0.0211 (2)	
N2	0.29562 (10)	0.29685 (13)	0.47526 (7)	0.0200 (2)	
N3	0.44949 (11)	0.40722 (14)	0.58388 (7)	0.0239 (3)	
C1	0.48140 (12)	0.26955 (16)	0.55234 (8)	0.0207 (3)	
C2	0.21931 (12)	0.23390 (16)	0.42367 (8)	0.0203 (3)	
C4	0.02092 (12)	0.24228 (16)	0.34674 (8)	0.0223 (3)	
C5	−0.08460 (12)	0.32173 (16)	0.32039 (8)	0.0223 (3)	
C3	0.10997 (11)	0.31895 (15)	0.39506 (8)	0.0191 (3)	
C6	−0.10163 (11)	0.47803 (16)	0.34134 (8)	0.0197 (3)	
C7	−0.01078 (12)	0.55645 (15)	0.38947 (8)	0.0192 (3)	
C8	0.09313 (12)	0.47800 (16)	0.41617 (8)	0.0191 (3)	
C9	0.51071 (12)	0.51201 (16)	0.63818 (8)	0.0212 (3)	
C10	0.61312 (13)	0.47347 (18)	0.68703 (8)	0.0254 (3)	
C11	0.66528 (13)	0.58702 (18)	0.73831 (8)	0.0266 (3)	
C12	0.61602 (13)	0.73693 (18)	0.74239 (9)	0.0261 (3)	
C13	0.51291 (13)	0.77472 (18)	0.69408 (9)	0.0276 (3)	
C14	0.46044 (13)	0.66315 (17)	0.64255 (9)	0.0247 (3)	
C15	0.05810 (13)	0.80047 (18)	0.44801 (9)	0.0254 (3)	
C16	−0.24398 (16)	0.9612 (2)	0.37071 (10)	0.0355 (4)	
H1	0.2343 (13)	0.1272 (19)	0.4021 (8)	0.021 (4)*	
H2	0.0297 (14)	0.131 (2)	0.3319 (9)	0.028 (4)*	
H3	−0.1460 (14)	0.269 (2)	0.2890 (9)	0.028 (4)*	
H4	0.1569 (14)	0.5318 (19)	0.4486 (9)	0.026 (4)*	
H5	0.6477 (14)	0.370 (2)	0.6856 (9)	0.030 (4)*	
H6	0.7391 (15)	0.5623 (19)	0.7720 (9)	0.032 (4)*	
H7	0.6504 (15)	0.818 (2)	0.7786 (10)	0.031 (4)*	
H8	0.4727 (15)	0.883 (2)	0.6956 (9)	0.032 (4)*	
H9	0.3903 (15)	0.688 (2)	0.6076 (10)	0.031 (4)*	
H10	0.0725 (14)	0.757 (2)	0.5024 (10)	0.029 (4)*	
H11	0.1347 (15)	0.7966 (19)	0.4238 (9)	0.027 (4)*	
H12	0.0276 (15)	0.910 (2)	0.4506 (9)	0.033 (4)*	
H13	0.3793 (16)	0.438 (2)	0.5638 (9)	0.030 (4)*	
H14	0.4096 (15)	0.115 (2)	0.4769 (10)	0.035 (5)*	
H15	−0.2449 (17)	0.513 (2)	0.2819 (11)	0.049 (6)*	
H16	−0.2038 (19)	0.922 (3)	0.4223 (14)	0.067 (6)*	
H17	−0.321 (2)	0.914 (3)	0.3632 (12)	0.064 (7)*	
H18	−0.2597 (19)	1.079 (3)	0.3773 (12)	0.061 (6)*	
H19	−0.1477 (18)	0.846 (3)	0.3092 (12)	0.052 (6)*	

### Atomic displacement parameters (Å^2^)

**Table:**

	*U*^11^	*U*^22^	*U*^33^	*U*^12^	*U*^13^	*U*^23^
S1	0.02158 (19)	0.0286 (2)	0.0418 (2)	0.00936 (14)	−0.00942 (15)	−0.01015 (16)
O1	0.0196 (5)	0.0227 (5)	0.0306 (6)	0.0036 (4)	−0.0071 (4)	−0.0031 (4)
O2	0.0194 (5)	0.0176 (5)	0.0300 (5)	0.0020 (4)	−0.0043 (4)	−0.0050 (4)
O3	0.0264 (5)	0.0231 (5)	0.0297 (6)	0.0045 (4)	−0.0013 (4)	0.0023 (4)
N1	0.0179 (6)	0.0204 (6)	0.0242 (6)	0.0045 (4)	−0.0020 (4)	−0.0017 (5)
N2	0.0173 (5)	0.0205 (6)	0.0220 (6)	0.0043 (4)	0.0000 (4)	0.0018 (5)
N3	0.0180 (6)	0.0229 (6)	0.0291 (6)	0.0048 (5)	−0.0061 (5)	−0.0042 (5)
C1	0.0190 (6)	0.0214 (7)	0.0214 (7)	0.0008 (5)	0.0008 (5)	0.0011 (5)
C2	0.0209 (7)	0.0190 (7)	0.0208 (7)	0.0014 (5)	0.0006 (5)	−0.0001 (5)
C4	0.0239 (7)	0.0177 (7)	0.0245 (7)	0.0011 (5)	−0.0010 (6)	−0.0016 (5)
C5	0.0212 (7)	0.0202 (7)	0.0240 (7)	−0.0013 (5)	−0.0043 (5)	−0.0015 (5)
C3	0.0184 (6)	0.0199 (7)	0.0187 (6)	0.0013 (5)	0.0005 (5)	0.0011 (5)
C6	0.0171 (6)	0.0210 (7)	0.0206 (7)	0.0019 (5)	−0.0006 (5)	0.0022 (5)
C7	0.0208 (6)	0.0158 (6)	0.0207 (7)	0.0002 (5)	0.0018 (5)	0.0000 (5)
C8	0.0177 (6)	0.0200 (7)	0.0191 (6)	−0.0003 (5)	−0.0005 (5)	−0.0005 (5)
C9	0.0199 (7)	0.0220 (7)	0.0216 (7)	−0.0013 (5)	0.0019 (5)	−0.0001 (5)
C10	0.0244 (7)	0.0237 (7)	0.0271 (7)	0.0033 (6)	−0.0029 (6)	−0.0011 (6)
C11	0.0235 (7)	0.0300 (8)	0.0251 (7)	−0.0009 (6)	−0.0028 (6)	−0.0010 (6)
C12	0.0264 (7)	0.0269 (7)	0.0255 (7)	−0.0059 (6)	0.0042 (6)	−0.0050 (6)
C13	0.0277 (7)	0.0238 (7)	0.0314 (8)	0.0022 (6)	0.0038 (6)	−0.0026 (6)
C14	0.0218 (7)	0.0251 (7)	0.0268 (7)	0.0038 (6)	0.0007 (6)	−0.0019 (6)
C15	0.0228 (7)	0.0218 (7)	0.0305 (8)	−0.0009 (6)	−0.0025 (6)	−0.0068 (6)
C16	0.0295 (9)	0.0420 (10)	0.0355 (9)	0.0070 (7)	0.0059 (7)	0.0097 (7)

### Geometric parameters (Å, º)

**Table:**

S1—C1	1.6832 (13)	C3—C8	1.4055 (18)
O1—C6	1.3630 (15)	C6—C7	1.4102 (18)
O1—H15	0.86 (2)	C7—C8	1.3753 (18)
O2—C7	1.3703 (15)	C8—H4	0.973 (16)
O2—C15	1.4308 (16)	C9—C10	1.3906 (19)
O3—C16	1.421 (2)	C9—C14	1.3964 (19)
O3—H19	0.83 (2)	C10—C11	1.391 (2)
N1—C1	1.3579 (17)	C10—H5	0.952 (17)
N1—N2	1.3766 (15)	C11—C12	1.382 (2)
N1—H14	0.896 (19)	C11—H6	0.983 (17)
N2—C2	1.2843 (17)	C12—C13	1.390 (2)
N3—C1	1.3440 (18)	C12—H7	0.975 (17)
N3—C9	1.4130 (17)	C13—C14	1.383 (2)
N3—H13	0.866 (18)	C13—H8	1.020 (17)
C2—C3	1.4597 (18)	C14—H9	0.964 (17)
C2—H1	0.993 (16)	C15—H10	1.004 (17)
C4—C3	1.3918 (18)	C15—H11	0.991 (17)
C4—C5	1.3925 (19)	C15—H12	0.983 (18)
C4—H2	0.975 (17)	C16—H16	1.01 (2)
C5—C6	1.3831 (19)	C16—H17	0.95 (2)
C5—H3	0.941 (16)	C16—H18	1.01 (2)
			
C6—O1—H15	109.5 (13)	C7—C8—H4	121.0 (9)
C7—O2—C15	116.58 (10)	C3—C8—H4	118.9 (9)
C16—O3—H19	108.7 (15)	C10—C9—C14	119.45 (13)
C1—N1—N2	119.57 (11)	C10—C9—N3	124.81 (13)
C1—N1—H14	119.2 (11)	C14—C9—N3	115.72 (12)
N2—N1—H14	121.2 (11)	C11—C10—C9	119.51 (14)
C2—N2—N1	116.69 (11)	C11—C10—H5	119.9 (10)
C1—N3—C9	132.57 (12)	C9—C10—H5	120.6 (10)
C1—N3—H13	111.3 (11)	C12—C11—C10	121.10 (14)
C9—N3—H13	116.0 (11)	C12—C11—H6	118.6 (10)
N3—C1—N1	114.01 (12)	C10—C11—H6	120.3 (10)
N3—C1—S1	127.69 (10)	C11—C12—C13	119.31 (14)
N1—C1—S1	118.30 (10)	C11—C12—H7	122.3 (10)
N2—C2—C3	120.52 (12)	C13—C12—H7	118.4 (10)
N2—C2—H1	120.5 (8)	C14—C13—C12	120.19 (14)
C3—C2—H1	118.9 (8)	C14—C13—H8	117.8 (9)
C3—C4—C5	120.31 (13)	C12—C13—H8	122.0 (9)
C3—C4—H2	121.1 (9)	C13—C14—C9	120.44 (13)
C5—C4—H2	118.6 (9)	C13—C14—H9	121.3 (10)
C6—C5—C4	120.41 (12)	C9—C14—H9	118.3 (10)
C6—C5—H3	119.0 (10)	O2—C15—H10	110.1 (9)
C4—C5—H3	120.5 (10)	O2—C15—H11	112.1 (9)
C4—C3—C8	119.39 (12)	H10—C15—H11	108.6 (13)
C4—C3—C2	119.98 (12)	O2—C15—H12	105.8 (10)
C8—C3—C2	120.62 (12)	H10—C15—H12	108.6 (14)
O1—C6—C5	123.84 (12)	H11—C15—H12	111.6 (14)
O1—C6—C7	116.85 (12)	O3—C16—H16	114.0 (13)
C5—C6—C7	119.30 (12)	O3—C16—H17	113.2 (13)
O2—C7—C8	125.07 (12)	H16—C16—H17	107.7 (18)
O2—C7—C6	114.46 (11)	O3—C16—H18	109.9 (12)
C8—C7—C6	120.46 (12)	H16—C16—H18	106.6 (18)
C7—C8—C3	120.10 (12)	H17—C16—H18	104.8 (18)
			
C1—N1—N2—N2	0.00 (10)	O2—O2—C7—C6	0.0 (4)
C1—N1—N2—C2	−179.57 (12)	C15—O2—C7—C6	−175.37 (12)
N2—N1—N2—C2	0 (33)	O1—C6—C7—O2	−1.49 (17)
C9—N3—C1—N1	−175.91 (13)	O1—C6—C7—O2	−1.49 (17)
C9—N3—C1—S1	3.6 (2)	C5—C6—C7—O2	177.86 (12)
N2—N1—C1—N3	4.88 (18)	O1—C6—C7—O2	−1.49 (17)
N2—N1—C1—N3	4.88 (18)	O1—C6—C7—O2	−1.49 (17)
N2—N1—C1—S1	−174.68 (10)	C5—C6—C7—O2	177.86 (12)
N2—N1—C1—S1	−174.68 (10)	O1—C6—C7—C8	179.85 (12)
N1—N2—C2—N2	0 (78)	O1—C6—C7—C8	179.85 (12)
N2—N2—C2—C3	0.00 (13)	C5—C6—C7—C8	−0.8 (2)
N1—N2—C2—C3	−178.52 (11)	O2—C7—C8—C3	−177.97 (12)
C3—C4—C5—C6	0.6 (2)	O2—C7—C8—C3	−177.97 (12)
C5—C4—C3—C8	−0.9 (2)	C6—C7—C8—C3	0.5 (2)
C5—C4—C3—C2	178.81 (13)	C4—C3—C8—C7	0.3 (2)
N2—C2—C3—C4	−171.87 (13)	C2—C3—C8—C7	−179.37 (12)
N2—C2—C3—C4	−171.87 (13)	C1—N3—C9—C10	−17.0 (2)
N2—C2—C3—C8	7.8 (2)	C1—N3—C9—C14	164.40 (15)
N2—C2—C3—C8	7.8 (2)	C14—C9—C10—C11	−1.3 (2)
O1—O1—C6—C5	0.00 (5)	N3—C9—C10—C11	−179.84 (13)
O1—O1—C6—C7	0.00 (7)	C9—C10—C11—C12	1.0 (2)
C4—C5—C6—O1	179.54 (13)	C10—C11—C12—C13	−0.3 (2)
C4—C5—C6—O1	179.54 (13)	C11—C12—C13—C14	0.0 (2)
C4—C5—C6—C7	0.2 (2)	C12—C13—C14—C9	−0.4 (2)
C15—O2—C7—O2	0 (24)	C10—C9—C14—C13	1.0 (2)
O2—O2—C7—C8	0.0 (4)	N3—C9—C14—C13	179.68 (13)
C15—O2—C7—C8	3.22 (19)	C7—O2—C15—O2	0 (76)

### Hydrogen-bond geometry (Å, º)

**Table:**

*D*—H···*A*	*D*—H	H···*A*	*D*···*A*	*D*—H···*A*
N3—H13···N2	0.866 (18)	2.082 (17)	2.5865 (16)	116.4 (14)
N1—H14···S1^i^	0.896 (19)	2.530 (19)	3.4033 (13)	165.2 (15)
O1—H15···O3^ii^	0.86 (2)	1.81 (2)	2.6562 (14)	167.7 (19)
O3—H19···O2	0.83 (2)	2.26 (2)	2.8853 (14)	132.1 (18)
O3—H19···O1	0.83 (2)	2.46 (2)	3.1645 (15)	144.2 (18)

Symmetry codes: (i) −*x*+1, −*y*, −*z*+1; (ii) −*x*−1/2, *y*−1/2, −*z*+1/2.
