# Supplementary figures and images for: Crystal structure of 4-hy­droxy-3-meth­oxy­benzaldehyde 4-methyl­thio­semi­carbazone methanol monosolvate
Source: Acta Crystallogr E Crystallogr Commun. 2015 Apr 18;71(Pt 5):o313–4. doi: 10.1107/S2056989015007227 (PMC4420048; doi:10.1107/S2056989015007227)

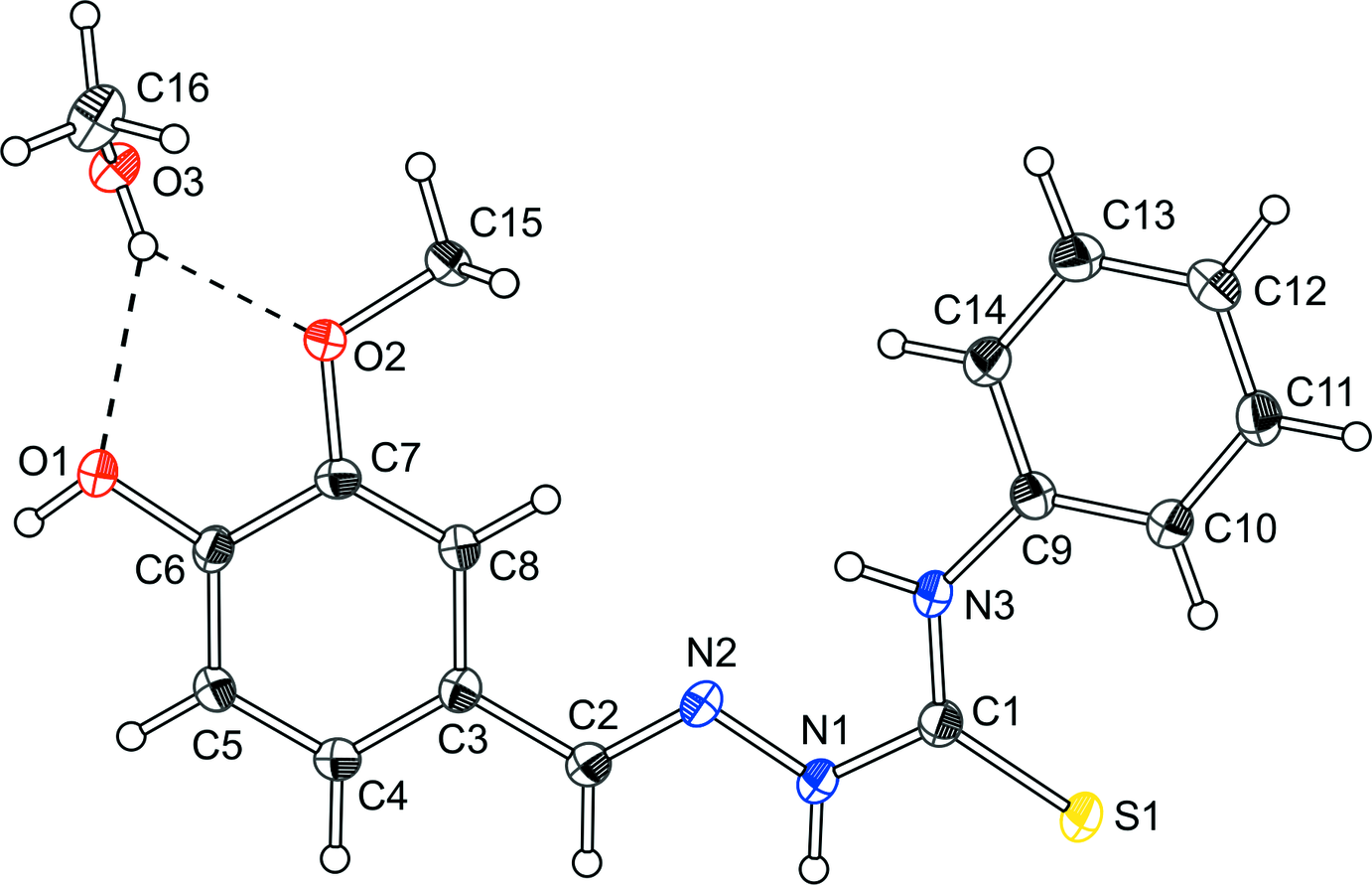

Supplement: Supplementary file 4 [file e-71-0o313-fig1.tif]

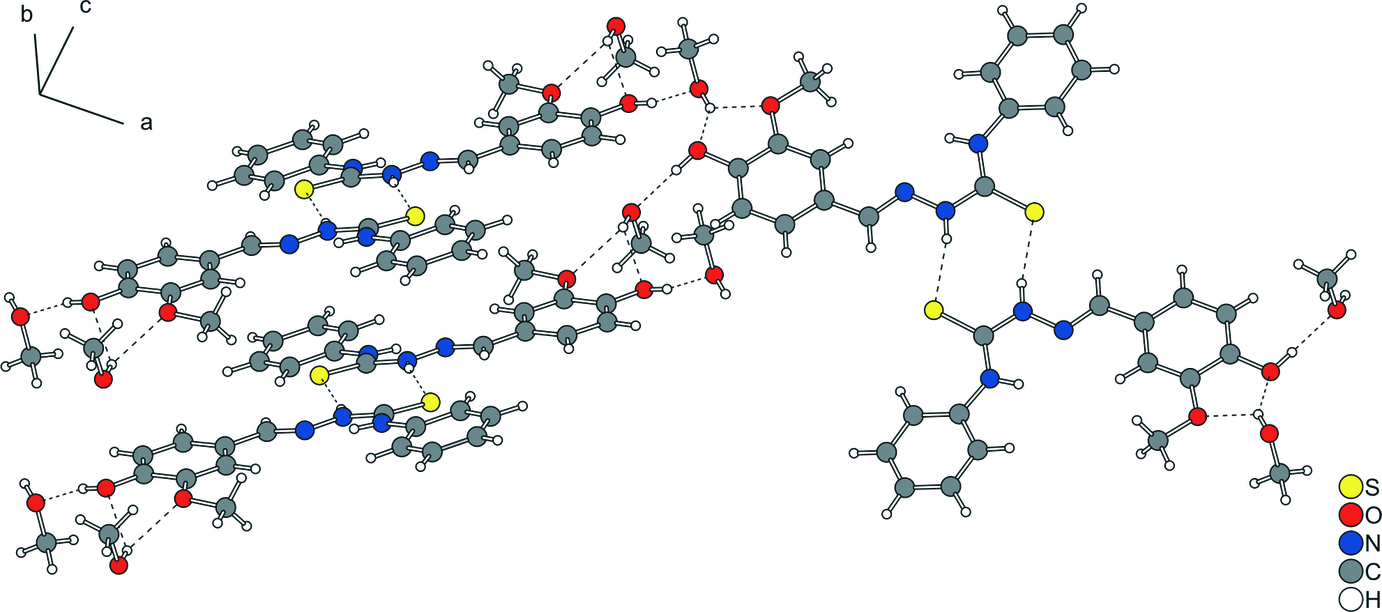

Supplement: Supplementary file 5 [file e-71-0o313-fig2.tif]
